# Invasive pulmonary aspergillosis in acute exacerbation of chronic obstructive pulmonary disease and the diagnostic value of combined serological tests

**DOI:** 10.4103/0256-4947.62828

**Published:** 2010

**Authors:** Xiaofang Gao, Liangan Chen, Guangrong Hu, Haiyu Mei

**Affiliations:** Form the Department of Respiratory Medicine, Chinese People's Liberation Army General Hospital, Beijing, Chinese People's Liberation Army No. 150 Hospital, Luoyang, China

## Abstract

**BACKGROUND AND OBJECTIVES:**

Invasive pulmonary aspergillosis (IPA) among patients with chronic obstructive pulmonary disease (COPD) is increasing in frequency. We conducted this study to find out the approximate incidence of IPA in patients with acute exacerbation of COPD (AECOPD), and to determine whether using a combination of two tests (galactomannan [GM] antigen and 1,3-β-glucan [BG] detection) would result in a more specific diagnosis of IPA.

**METHODS:**

The study included 261 patients with AECOPD admitted over two years. The patients were categorized according to the modified definitions for IPA. GM antigen and BG were detected by the Platelia *Aspergillus* and Glucatell tests.

**RESULTS:**

Two patients had proven IPA, three had probable IPA, and two had possible IPA. The rate of proven or probable IPA in patients with AECOPD was 1.91% (5/261). Four patients with proven and probable IPA had been treated with a systemic or inhaled corticosteroid before hospitalization and the typical symptoms and diagnostic signs of IPA were relatively less common in them. Mortality in patients with proven and probable IPA was 80%. The specificity of combined GM and BG detection was 98.8%.

**CONCLUSION:**

Combining two serological tests increased the specificity of diagnosis but further trials are needed to prove the value of this approach.

Over the last decade, the incidence of invasive aspergillosis (IA) has significantly increased in neutropenic patients and matched unrelated donor transplant recipients.^1^ This severe opportunistic fungal infection is characterized by a high mortality rate in these at-risk patients.^2^ Several publications indicate that the population at risk for pulmonary or disseminated IA should be expanded to include patients with chronic obstructive pulmonary disease (COPD).^3^,^4^

A systematic review showed that among 1941 patients with IA, 26 patients (1.3%) had COPD.^5^ In one large study, 9% of 595 patients with IA suffered from some form of pulmonary disease.^6^ Rodrigues et al reported that COPD patients constituted 1% of all cases of IA at their institution.^7^ Recently, Guinea et al reported that invasive pulmonary aspergillosis (IPA) affected at least 22.1% of patients with COPD and that *Aspergillus* could be isolated by culture.^8^ Although these numbers are impressive, the true incidence of IPA in COPD is probably underestimated, especially in a high-risk subset of patients, for example, those with acute exacerbation of COPD (AECOPD).

Establishing a diagnosis of IPA remains a challenge. Cultures may require days or weeks to grow, while the invasive procedures needed to obtain specimens for histopathological examination can rarely be performed on many patients. In the past decade, noninvasive diagnostic tests have focused on the detection of surrogate markers such as the galactomannan (GM) antigen and 1,3-β-glucan (BG). One study showed that the determination of GM in the sera of non-neutropenic patients could prove useful for diagnosing IA.^9^ However, the use of these new diagnostic tools is hampered by a high rate of false-positive results.^10^ The aim of this study was to find the approximate incidence of IPA in patients with AECOPD and whether a combination of two tests-GM and BG detection-would result in a more specific diagnosis of IPA.

## METHODS

We performed this prospective study from October 2006 to November 2008 at No. 150 Hospital of the Chinese People's liberation Army (PLA), China. There are 1100 beds in this hospital, with 60 beds in the respiratory diseases department. All patients diagnosed with AECOPD during the study period were included. Patients with AECOPD were classified according to Global Initiative for Chronic Obstructive Lung Disease (GOLD) criteria.^11^ The following data were collected for each patient: age, gender, accompanying disease, details of steroid treatment and broad-spectrum antibiotic received, history of mechanical ventilation, peripheral white blood cell count, radiographic findings, and frequency of hospitalization during the previous year, hospitalization duration, and outcome. Respiratory specimens were cultured on conventional medium, and selected specimens were further cultured on fungal media for at least 7 days. *Aspergillus* was identified by standard morphological procedures. A blood sample of each patient was obtained after the preliminary diagnosis had been made. Serum samples were stored at −20°C until use. This study was performed with the approval of the Ethics Committee of the Chinese People's Liberation Army No. 150 Hospital. Informed consent was obtained from all study subjects.

The EORTC/MSG (European Organization for the Treatment of Cancer/Mycoses Study Group) guidelines for IPA were not designed for patient categories other than cancer patients and bone marrow transplant recipients.^1^ One important at-risk group, patients with COPD, was not included in this definition. So we used modified IPA definitions to classify our patients as follows:^1^,^3^,^4^,^12^–^14^

- ‘Proven IPA’ referred to those who had histopathologic or cytopathological evidence of tissue invasion (in needle aspiration or biopsy and/or autopsy specimen), with septated, acutely branching hyaline hyphae compatible with *Aspergillus* seen on microscopy, or culture of a sample from a normally sterile but clinically infected body site (excluding bronchoalveolar lavage fluid and sinus aspirate) obtained by a sterile procedure was positive for *Aspergillus* species.
- ‘Probable IPA’ referred to those with positive culture results or cytological evidence for *Aspergillus* species from a lower respiratory tract (LRT) specimen, in conjunction with one major sign (halo sign or ‘air crescent’ sign on CT scan) or at least two minor signs (LRT infection, pleural rub, and presence of any new infiltrate in a patient who did not fulfill the major criterion but for whom no alternative diagnosis was available). ‘Probable IPA’ was also applied to the COPD patient, usually treated with steroids, that had severe disease (stage III or IV) according to GOLD criteria, with recent exacerbation of dyspnea resistant to appropriate treatment (including antibiotics) and with suggestive chest imaging (pulmonary lesions shown by radiograph or CT scan to be unresponsive to appropriate antibiotics), accompanied by one of the following: i) Positive culture and/or microscopy for *Aspergillus* from LRT, ii) positive serum antibody test for *Aspergillus* fumigatus, iii) two consecutive positive serum GM tests.
- ‘Possible IPA’ referred to the COPD patient, usually treated with steroids, that had severe disease according to GOLD criteria (stage III or IV), with recent exacerbation of dyspnea that was resistant to appropriate treatment (including antibiotics) and with suggestive chest imaging, but without positive *Aspergillus* culture or microscopy from LRT or serology.
- ‘Colonization’ referred to those with positive culture for *Aspergillus* from a nonsterile site, but without any other evidence of fungal infection.

The detection of GM antigen by the Platelia *Aspergillus* EIA test (Bio-Rad Laboratories, Marnes, France) was carried out according to the manufacturer's instructions. A sample was considered positive if the index was ≥1.5.^15^ BG was detected with the Glucatell test kit (Associates of Cape Cod, Falmouth, Massachusetts, US). Patients were judged positive if the level of BG was ≥80 pg/mL.^16^ Testing for GM antigen and BG was done in batches. If the patient was still in hospital when the serology result was reported, the treating physician clinician received the result.

Sensitivity, specificity, and positive and negative predictive values were calculated as described by Kozinn.^17^ According to Mennink-Kersten et al, only proven and probable IPA were considered truly positive and only no IPA cases were considered truly negative. Patients classified as possible IPA were not included in the analyses because of diagnostic uncertainty.^18^

## RESULTS

The study included 261 patients with AECOPD who were admitted during the study period (October 2006 to November 2008). The mean (SD) age of patients was 65.3 (9.6) years. According to the GOLD severity classification, 105 (40.2%) patients were in stage I and II and 156 (59.8%) in stage III and IV. Seventy-one (27.2%) patients had a history of treatment with systemic or inhaled corticosteroid in the 3 months preceding admission, and 87 (33.3%) patients had been admitted on at least one occasion during the previous year. Among these patients, 2 had proven IPA, 3 had probable IPA, and 254 patients did not have IPA; the remaining 2 patients had possible IPA.

For the five IPA patients (proven and probable IPA), the main clinical sign was nonspecific antibiotic-resistant pneumonia associated with exacerbation of dyspnea. On hospital admission, four IPA patients had been treated with corticosteroids, and three received an accumulated dose (or equivalent) of prednisone of >700 mg over the last 3 months. All IPA patients were administered systemic corticosteroids during hospitalization, and four received a prednisone equivalent of >0.3 mg/kg/day. Among non-IPA patients (possible and no IPA), 28 (10.9%) and 39 (15.2%) patients, respectively, had a history of receiving systemic or inhaled corticosteroid before admission; 52 (20.3%) patients received systemic corticosteroid during hospitalization, and 16 (38.1%) received a prednisone equivalent >0.3 mg/kg/day.

In contrast to what is seen in neutropenic patients and stem cell transplant recipients, the relatively typical symptom of fever was present in only two patients.^19^ Chest pain and hemoptysis also were present in only two IPA patients. Thoracic CT scan was performed on 4 IPA patients and 102 non-IPA patients, but the diagnostic signs—the halo sign or the air-crescent sign—were only found in only 1 IPA patient. On the whole, there were no significant differences in the CT scan findings of IPA and non-IPA patients. *Aspergillus* culture was positive in four IPA patients and two non-IPA patients (colonization).

Nearly all non-IPA patients (251/254; 98.8%) received antibiotic treatment, with the duration ranging from 5 days to 42 days. Three IPA patients received antibiotic treatment, the mean duration being 12.3 days. Twenty-three non-IPA patients (9.1%) received piperacillin–tazobactam or amoxicillin–clavulanic acid, both of which can interfere with GM detection. Eighteen non-IPA patients (7.1%) had an associated non-aspergillosis infection (usually *Candida*), which can interfere with BG detection. All IPA patients received voriconazole or caspofungin, and three non-IPA patients received anti-*Aspergillus* therapy. The mortalities were 80.0% (4/5) and 5.1% (13/256) in IPA and non-IPA patients, respectively. The main causes of death in these two groups were respiratory failure (IPA: 3/4; non-IPA: 7/13), uncontrolled infection (IPA: 1/4; non-IPA: 4/13) and sepsis (IPA: 0/4; non-IPA: 2/13). The sample size was too small to carry out univariate analysis or multivariable logistic regression analysis. According to the characteristics of the patients, the possible risk factors for IPA in patients with AECOPD were corticosteroid use, mechanical ventilation, and the frequency of hospital admissions in the preceding year (Table 1).

**Table 1:** Characteristics of the five patients with proven or probable IPA.

Patients	1	2	3	4	5
IPA classification	Proven	Proven	Probable	Probable	Probable
Gender	Male	Male	Female	Male	Male
Age (years)	65	71	73	63	67
Smoker	Yes	Yes	No	Yes	Yes
GOLD classification	Stage III	Stage IV	Stage III	Stage III	Stage IV
Accompanying disease	Hypertension	Renal inadequacy	Diabetes mellitus	None	Heart failure
Steroid treatment at admission	Systemic	Systemic	Systemic	None	Inhaled
Hospital admissions in last year	2 times	1 time	2 times	1 time	3 times
Thoracic CT scan	Infiltration	Halo sign	Cavitation	Infiltration	Not done
Days between admission and confirmed diagnosis	6	5	12	18	16
Ventilation	Invasive	Invasive	Invasive	None	invasive
WBC count	14.3×10^9^/L	9.1×10^9^/L	13.6×10^9^/L	8.6×10^9^/L	12.7×10^9^/L
Sampling methods for *Aspergillus*	Bronchial biopsy by bronchoscopy	Lung biopsy by percutaneous lung puncture	Culture from sputum	Culture from LRT	Culture from LRT
Outcome	Death	Death	Death	Survival	Death

Of 261 samples obtained from the subjects, the test for GM was positive in 20 patients and negative in 241 patients. A GM-positive result was obtained in one patient with possible IPA. The positive predictive value (PPV) and negative predictive value (NPV) were 21.1% and 99.6%, respectively. The test for BG was positive in 48 patients and negative in 213 patients. Both of the two patients with possible IPA had positive results. The PPV and NPV were 8.7% and 99.5%, respectively. There was one proven IPA patient who tested negative for both GM and BG; this patient had received large doses of corticosteroids and short-term anti-aspergillosis therapy before admission.

Most patients without IPA who were false positive for one of the seromarkers tended to be negative for the other one. We examined how combining the results of both tests helped in diagnosis of IPA. IPA was diagnosed only when both seromarkers were positive in any one patient. When both markers were used in combination there was an improvement in diagnostic specificity (Table 2).

**Table 2:** Performance of galactomannan [GM] antigen and 1,3-β-glucan [BG] and the combination in detection of invasive aspergillosis.

Test	No. of results				Sensitivity	Specificity
	True positive	True negative	False positive	False negative		
GM detection	4	239	15	1	80%	94.1%
BG detection	4	212	42	1	80%	83.4%
Combination	4	251	3	1	80%	98.8%

## DISCUSSION

Assessing the incidence of IPA in relatively less immunocompromised patients (patients with AECOPD for example) is not easy because of the lack of a consistent case definition and the absence of a perfect surveillance system. We used a modified definition of IPA for diagnosis, which resulted in rate of proven or probable IPA in patients with AECOPD of 1.91% (5/261), which suggests the possibility of considering IPA as a differential diagnosis in advanced COPD patients. According to the characteristics of the IPA and non-IPA patients in the present study, the increased susceptibility to invasive *Aspergillus* infection might be caused by long-term or repeated short-term corticosteroid treatment, broad-spectrum antibiotic treatment, and the presence of comorbidities such as diabetes mellitus and renal inadequacy.

During the acute exacerbation period of COPD, a systemic or inhaled corticosteroid is recommended to be used to relieve bronchospasm and the systemic inflammatory response syndrome.^20^ However, corticosteroids are a double-edged sword since they can impair immune function and have been shown to directly stimulate the growth of *A fumigatus* in vitro.^21^ Some reports have suggested that not only systemic, but inhaled corticosteroids might also promote IPA in COPD patients.^22^ One patient diagnosed as probable IPA in the present study had received only inhaled corticosteroids. The present study also showed that the frequency of administration of systemic corticosteroids in IPA patients was much higher than in non-IPA patients (100.0% vs. 20.3%), as was the dosage of corticosteroid. This indicates that under certain circumstances clinicians should use corticosteroids with caution.

The clinical picture of IPA in patients with AECOPD was very nonspecific. The signs and symptoms suggestive of angioinvasion were usually absent. In the present study more than half of the cases did not have fever. Of the IPA patients who underwent thoracic CT scan, only one patient had the halo sign, which is consistent with the reported lower sensitivity (5%-24%) of the halo and air crescent signs in non-neutropenic patients in the literature.^23^ The absence of the halo sign in this population had no negative predictive power, and if the signs were present, they were far less specific.

The mortality of IPA patients in the present study reached 80%, whereas the mortality was only 5.1% among non-IPA patients, which is in accordance with the findings of Rello et al.^4^ The high mortality urged us to find a practical method for early diagnosis of IPA in patients with AECOPD. GM and BG are polysaccharide fungal cell wall components that are released during tissue invasion and can be detected in specimens of body fluids.^24^ They are considered useful surrogate markers for invasive fungal infection.^14^ The overall sensitivity of both GM and BG in the present study was 80%, which was in agreement with the result reported in literature.^15^ The lower PPV and higher NPV in the present study compared with other studies were caused by the relatively low prevalence of IPA in the population with AECOPD. However, the use of GM and BG detection in clinical practice is hampered by high rates of false positive results. In our study, there were 15 and 42 false-positive results with GM and BG, respectively. False-positive BG reactions were associated with fungal infection (non-aspergillosis), the use of albumin, hemodialysis, and certain bacterial infections.^25^ False-positive reactions with GM might be caused by the use of piperacillin-tazobactam, amoxicillin-clavulanic acid, or antifungal drugs.^9^ A strategy to overcome the inherent deficiencies of these serological tests could be the use of a combination of the surrogate markers of IPA. The results of this study suggest that the combination of BG and GM detection for confirming the existence of IPA was more specific than either test used alone. The value of a combination of GM and BG detection deserves further study.

In conclusion, IPA in patients with AECOPD is an emerging problem. The use of corticosteroids plays an important role in its pathogenesis. IPA poses a major threat to this group of patients, and the associated mortality can be devastating. The combination of BG and GM detection is useful for identifying the false-positive reactions by any one test used alone. The value of this approach needs to be proven in future trials.
